# Sport doping detection in focus: A perspective from Prof. Dr Mario Thevis

**DOI:** 10.1002/ansa.202000022

**Published:** 2020-05-12

**Authors:** Mario Thevis, Paul Trevorrow

**Affiliations:** ^1^ Center for Preventive Doping Research, Institute of Biochemistry German Sport University Cologne Cologne Germany; ^2^ John Wiley and Sons Ltd., The Atrium, Southern Gate Chichester Po19 8SQ UK

## 1 WOULD YOU BRIEFLY EXPLAIN WHAT YOUR RESEARCH GROUP IS STUDYING?

Our group focuses on antidoping research in many of its facets, and that includes new drugs and drug candidates concerning testing strategies and metabolic conversion of the substances. We are aiming at developing test methods or improving existing approaches in the light of new technologies, and we investigate alternatives to complement conventional doping tests, for instance, by means of matrices complementary to serum and urine.

## 2 THE ANTIDOPING EXPERT HAS TO MAINTAIN A BROAD SET OF ANALYTICAL SKILLS COULD YOU DESCRIBE SOME OF THE ROUTINE TECHNIQUES (AS WELL AS SOME MORE EMERGENT TECHNIQUES THAT YOU USE?)

That is the particularly interesting aspect of antidoping research and routine analysis: the breadth of analyzed and employed strategies. But it is, at the same time, a considerable challenge, financially and with regards to space and qualified personnel. The predominant technology used is mass spectrometry complemented by immunological and electrophoretic testing approaches. About 95% of all target analytes are covered by mass spectrometry. In addition, since the list of prohibited substances and methods is in some instances not limited to the presented examples of banned compounds, we exploit what is referred to as the digital matrix of doping control samples. Once a specimen is analyzed by, for instance, high‐resolution mass spectrometry, the data can be reprocessed for various additional analytes that might not have been covered during the first screen, when for example formerly unknown co‐called designer substances were suspected.

## 3 WHAT ARE YOUR VIEWS ON THE FUTURE AND CHALLENGES OF ANTIDOPING?

We do face quite a variety of challenges. This includes; increasing the test spectrum, faster turnaround times, and certainly reduced costs. It is particularly complex, spanning from inorganic transition metal analysis and gaseous substances via natural and synthetic steroids and stimulants, and so on, to extremely short‐lived peptides and high molecular mass proteins. All of those analytes have to be tested from a limited volume of urine or serum, mostly at extremely low levels. Having said that, our assays have inevitably been developed for utmost sensitivity and specificity. That is for the result managing authorities and, to some extent, the athletes both friend and foe at the same time. The retrospectivity has greatly improved. In other words, the periods covered by the tests are longer than years ago and these have allowed test frequencies for athletes to be arranged at acceptable levels. The analytical sensitivity however also picks up contamination scenarios. Occasionally, an inadvertent uptake of banned substances, at most likely pharmacologically irrelevant concentrations, is the result of today's so‐called exposome. Deciding whether or not to impose a sanction on an athlete based on an adverse analytical finding is, in those cases, particularly difficult. Therefore, supporting result managing authorities with scientific data that indicate the time and amount of a potentially inadvertently administered drug, as well as scenarios of the intake itself, has become a priority project in my group.

## 4 IF YOU HAD A WISH LIST OF IMPROVEMENTS FOR THE ANALYTICAL CAPABILITIES OF TODAY WHAT WOULD THEY BE?

The instrumentation is already very good, but more dedicated analytical systems would be desirable as the necessity to combine as many target analytes into one single assay for the sake of the already mentioned cost‐effectiveness and speed is particularly challenging.

## 5 ARTIFICIAL INTELLIGENCE (AI) IS A MUCH‐USED BUZZWORD THESE DAYS, WHERE DO YOU SEE THIS INFLUENCING THE DETECTION WORKFLOW?

I do certainly see a role of AI in sports drug testing in the near future, and that applies especially to automated data review. Manual data evaluation is critical and under the above‐mentioned circumstances, still the most reliable approach. If technology such as AI‐based software can catch up and prove as reliable in data evaluation as an experienced analyst, AI would be a great complement to future doping control programs.

